# Leveraging Language Models for Automated Label Generation in Traumatic Brain Injury Radiology Reports

**DOI:** 10.21203/rs.3.rs-8051129/v1

**Published:** 2025-12-04

**Authors:** Lingrui Cai, Craig Williamson, Andrew Nguyen, Emily Wittrup, Kayvan Najarian

**Affiliations:** 1 Gilbert S. Omenn Department of Computational Medicine and Bioinformatics, University of Michigan, Ann Arbor, MI, USA; 2 Department of Neurosurgery and Neurology, University of Michigan, Ann Arbor, MI, USA; 3 Michigan Institute for Data Science, University of Michigan, Ann Arbor, MI, USA; 4 Max Harry Weil Institute for Critical Care Research and Innovation, University of Michigan, Ann Arbor, MI, USA

**Keywords:** Traumatic Brain Injury, Radiology Report, Nature Language Processing, Domain Adaptation, Semi-supervised Learning, Clinical Decision Support

## Abstract

Timely interpretation of head CT scans is critical for managing traumatic brain injury (TBI), yet delays in radiology reporting can slow urgent clinical decisions. To address this challenge, we developed natural language processing (NLP) frameworks that automatically convert free-text radiology reports into structured, machine-readable findings. Using 4,038 de-identified head CT reports, including 444 expert-annotated samples, we compared several strategies for improving clinical finding and location model accuracy. A lexicon-weighted domain-adaptive pretraining approach, designed to emphasize key diseased-related terms, achieved the best overall performance, reaching a weighted F1-score of 0.92 across five-fold cross-validation. A location-aware cascade model further improved recognition of anatomical sites, enhancing transparency and clinical relevance. Semi-supervised learning using unlabeled reports produced moderate gains over standard supervised models. These methods demonstrate that domain-specific adaptation and structured modeling can reliably extract critical findings from radiology text, enabling faster, more consistent interpretation of radiology reports. By enabling automated report generation from imaging and multimodal data, this framework may help shorten reporting turnaround times and facilitate more data-driven neurotrauma care.

## 1 Introduction

Traumatic brain injury (TBI) is a leading cause of morbidity and mortality worldwide, with an estimated 50 million new cases annually^1^. TBI encompasses a spectrum of neurological insults caused by external mechanical forces, resulting in substantial societal and economic burden due to acute care costs and long-term disability^2^. In the United States alone, TBI-related emergency department visits, hospitalizations, and deaths exceed 2.8 million annually, with falls representing the most common mechanism, particularly among older adults^3,4^. The pathophysiology of TBI involves primary injury (e.g., hematomas, contusions, diffuse axonal injury) and secondary injury cascades that may be exacerbated by modifiable systemic factors such as hypoxia, elevated intracranial pressure, and hypotension^5,6^. Rapid identification of key radiologic findings such as subdural hemorrhage, intraparenchymal bleeding, and midline shift is critical for triage and treatment decisions^7,8^.

Head computed tomography (CT) remains the recommended imaging modality for acute TBI evaluation, particularly in emergency settings where rapid detection and localization of hematomas are essential^8,9^. The accompanying radiology reports provide rich clinical context, describing the presence, characteristics, and anatomical distribution of injuries^10^. However, free-text reporting introduces ambiguity, variability among radiologists, frequent use of negations and hedging, and comparative statements with prior examinations, which complicate automated extraction of structured labels^11,12^. The lack of standardized terminology and the narrative emphasis on clinical reasoning, rather than categorical annotation, further hinder consistent mapping to structured data fields^13^. As a result, converting free-text reports into reliable structured information remains non-trivial and limits their use in large-scale computational analyses and downstream predictive modeling.

Radiology reports are increasingly used not only as clinical documentation but also as surrogate labels to build imaging models, report generation models, and vision–language models^14–16^. Compared with manual image annotation, which is resource-intensive and requires expert neuroimaging knowledge, report-derived labels offer scalability. Nevertheless, expert oversight is still needed, and annotation remains a bottleneck. Robust natural language processing (NLP) methods could enable reliable label extraction, reducing costs, enhancing reproducibility, and supporting large-scale datasets for comprehensive TBI AI research.

Transformer-based language models, including encoder-based models such as Bidirectional Encoder Representations from Transformers (BERT) and large language models (LLMs), have advanced clinical text understanding^17–19^. For domain adaptation, two principal strategies have emerged: domain-adaptive pretraining (DAPT), which continues pretraining of a general model on a domain corpus such as BioBERT^20^), and training from scratch (TFS) on domain text such as PubMedBERT^21^. The first approach leverages general linguistic knowledge before specialization, whereas the second learns domain semantics directly from biomedical corpora. Further specialization focuses on text type, for example, BioClinBERT integrating MIMIC-III clinical notes^22^; RadBERT trained on radiology reports^23^. Architectural enhancements such as DeBERTa’s disentangled attention^24^ and BioLinkBERT’s knowledge-linking^25^ also improve contextual representation(Table S1 for more details). Beyond encoders, open-weight LLMs, including Gemma^26^, Mistral^27^, LLaMA^28^, Qwen^29^, and their biomedical derivatives, for instance, BioMistral^30^, Me-LLaMA^31^, MedAlpaca^32^, have been adapted for the medical context via continued pre-training and instruction tuning to handle complex generative and reasoning tasks such as medical question answering (QA), clinical dialogue, text summarization, and diagnostic reasoning (more details in Table S2). Despite these advances, LLM deployment in specialized clinical environments poses challenges. Large parameter counts often require proprietary APIs or distributed computing, limiting local fine-tuning on institutional data. Moreover, patient privacy and HIPAA compliance remain critical when model inference involves transmitting clinical text externally. Recent work on parameter-efficient fine-tuning (PEFT), such as Low-Rank Adaptation (LoRA)^33^, aims to mitigate these issues by introducing lightweight, trainable parameters within frozen transformer layers, enabling efficient domain adaptation with reduced computational and privacy constraints.

Extracting anatomical location information from clinical narratives is essential for interpreting radiologic findings, where laterality, symmetry, and regional specificity hold diagnostic importance. Early systems such as SemRep^34^ and MedLEE^35^ relied on grammar-based rules and medical ontologies to identify location relations among biomedical concepts. More recent biomedical NLP models, such as BiLSTM^36^ and CoQAUD^37^, leverage LSTM and Transformer architectures to capture contextual dependencies and handle noisy clinical text. Advances in LLMs, such as ChatGPT^38^, further demonstrate strong capability in understanding complex spatial and relational contexts in radiology reports. However, few studies have focused on the joint modeling of identified findings and their anatomical locations, which is a critical aspect for comprehensive contextual interpretation and clinical utility.

Semi-supervised learning (SSL) approaches have been developed to address limited labeled data in model development. Common strategies include pseudo labeling^39^, consistency regularization^40^, and mean teacher model^41^. In biomedical applications, SSL has been applied to named entity recognition(NER) and clinical text classification with mixed success, often constrained by domain-specific language, label imbalance, and a lack of robust data augmentations^42^. Within the radiology domain, most studies have focused on self-supervised pretraining or weak supervision using report-derived labels^43^, whereas the application of consistency-based SSL remains largely unexplored. This gap highlights the need for domain-aware consistency and regularization strategies that can effectively leverage the unique linguistic structure of unlabeled radiology text while preserving essential clinical semantics.

Here, we present a unified framework for structured label extraction from TBI head CT radiology reports. Our contributions are threefold: (i) a comprehensive evaluation of encoder and generative architectures for multi-label findings classification; (ii) domain-adaptive pretraining with a lexicon-weighted masked language modeling objective tailored to a curated TBI lexicon, plus a two-stage cascade for anatomical localization; and (iii) a consistency-based SSL approach to leverage unlabeled reports. To our knowledge, this is the first study to combine comprehensive multi-label TBI finding extraction, lexicon-weighted DAPT, and cascade-based localization in a single pipeline, offering practical guidance for scalable auto-labeling in clinical NLP.

## 2 Results

### 2.1 Supervised findings classification

Domain-adaptive pretraining improved multi-label classification across encoder backbones (Table 1, Fig. 2). Both uniform MLM (*TBI-*) and lexicon-weighted MLM (*WTBI-*) variants outperformed their baselines. The strongest results were achieved by WTBI-PubMedBERT (weighted F1 = 0.92) and WTBI-BioLinkBERT (weighted F1 = 0.91). Gains were largest for lower-prevalence findings, including IPH and IVH. TBI-DeBERTa and TBI-PubMedBERT also improved IVH, and CFF increased reliably under pretraining, whereas SDH and SAH changed little given high baselines. Comparing *TBI-* with *WTBI-*indicates that emphasizing a curated TBI lexicon yields additional gains on terminology-dependent labels (IPH, IVH, CFF, MLS).

Fold-wise paired tests (Fig. 2) showed significant improvements (*P* < 0.05) for IPH, IVH, MLS, and CFF; effects for SDH and SAH were mixed due to strong baselines. Corpus alignment emerged as a key driver: literature-pretrained models such as PubMedBERT and BioLinkBERT, realized the largest relative gains after radiology-specific adaptation, while RadBERT which already trained on radiology text showed limited improvement. DeBERTa also benefited particularly for IVH and MLS. LoRA-tuned LLMs were competitive but below the best encoders. Mistral achieved weighted F1 = 0.87 (Table S6) as the top LLM, with the largest gaps on IVH and CFF, suggesting targeted encoder DAPT better captures radiology-specific cues with limited labeled data.

### 2.2 Location Classification with the Cascade Model

The cascade framework mirrors radiologists’ reasoning by first identifying a pathology and then classifying its anatomical location when relevant. Incorporating hierarchical loss improved F1-scores across all backbones and lesion types compared with flat, single-stage baselines. As shown in Table 2 and Figure 3, hierarchical models (Hier-) achieved average gains of 3–6 percentage points, with the largest improvements in low-prevalence, laterality-specific findings such as IPH(L) and IPH(R).

Among all encoders, Hier-PubMedBERT and Hier-BioLinkBERT achieved the highest performance , with a F1 score 0.93 for SDH(R) and F1 score 0.78 for IPH(R), followed closely by Hier-DeBERTa. Statistical comparisons showed significant gains (*p* < 0.05) for SDH(R), SAH(R), and IPH(R). Overall, the cascade model effectively captured hierarchical relationships between findings and spatial descriptors, enhancing interpretability and providing clinically meaningful laterality information for radiology report analysis in TBI.

### 2.3 Findings Classification with Semi-Supervised Learning

We further evaluated whether leveraging unlabeled reports through semi-supervised learning (SSL) could enhance classification performance. As shown in Table 3 and Figure 4, SSL models consistently improved weighted F1-scores compared with their fully supervised counterparts, particularly for low-prevalence findings such as IPH, IVH, and CFF. The largest relative gains were observed for DeBERTa, Biolink, and PubMedBERT, reaching weighted F1-scores of 0.894, 0.888, and 0.876, respectively. Among these, DeBERTa achieved the best overall performance, with significant improvement on IPH (*p* < 0.05).

Semi-supervised training also reduced performance variance across folds, suggesting improved stability and generalization. Gains were most evident for underrepresented or ambiguous findings, whereas common classes such as SDH and SAH changed minimally due to their high baselines. In general, SSL effectively utilized unlabeled radiology reports to enhance model robustness in data-limited settings, demonstrating its potential as a complementary strategy to domain-adaptive pretraining for large-scale clinical NLP applications.

## 3 Discussion

This study systematically evaluated multiple strategies to enhance automated extraction of traumatic brain injury (TBI) findings from radiology reports, including domain-adaptive pretraining with lexicon-weighted masked language modeling, semi-supervised learning with consistency regularization, and cascade modeling for anatomical location inference. Among these, domain-adaptive pretraining with lexicon weighting achieved the strongest and most consistent performance, particularly for rare or linguistically ambiguous findings such as intraparenchymal and intraventricular hemorrhage. By emphasizing clinically meaningful terms, the weighted objective improved contextual representation and label separability within the specialized linguistic space of radiology reporting. The integration of location-aware modeling provided complementary interpretive value by linking lesion descriptions to laterality and regional context. This addition improved classification for findings with spatially dependent meanings and enhanced model interpretability.

Despite the conceptual advantages of utilizing large corpora of unlabeled reports, the semi-supervised learning approach yielded only marginal improvements and did not exceed the performance of fully supervised, domain-pretrained models. Several factors may explain this outcome. The FixMatch-style consistency regularization used here was originally designed for image classification and may not optimally capture the complexity of clinical language in multi-label text settings. Additionally, pseudo-label noise, especially for low-prevalence findings, can attenuate the consistency signal and limit performance gains. Future work could explore hybrid teacher–student architectures or self-training frameworks better tailored to the structure of radiology text.

From a clinical perspective, improving automated classification of TBI findings has direct implications for acute and critical care decision support. Accurate extraction of hemorrhage type, midline shift, and fracture descriptions enables large-scale phenotyping and outcome modeling without the need for manual chart review. The weighted pretraining approach aligns model attention with clinically salient terminology, reducing the likelihood of errors in rare but critical cases, while the incorporation of anatomical location cues enhances interpretability and facilitates integration with imaging-based models or EHR systems. Together, these advances contribute toward an integrated, data-driven framework for timely and standardized TBI assessment.

Beyond modeling performance, metadata analysis (Table S3) underscores the practical need for more efficient imaging-to-report workflows. For the dataset involved in this study,each TBI admission involved an average of four CT scans, with cumulative reporting delays exceeding five hours from image acquisition to finalized interpretation. In time-sensitive scenarios, such as intracranial pressure management or surgical triage, these delays can affect patient outcomes. Automated findings classification and structured reporting could serve as intermediary tools for near real-time prioritization, assisting radiologists in high-volume settings and improving the timeliness and consistency of documentation.

Although the dataset in this study originates from a single academic institution, it represents a geographically and demographically diverse patient population across the state of Michigan. As a major referral center, Michigan Medicine receives patients from urban, suburban, and rural regions, and the involvement of numerous radiologists and clinicians ensures diversity in report structure, vocabulary, and diagnostic phrasing. This heterogeneity contributes to linguistic diversity despite the single-center origin, supporting the generalizability of the model.

We acknowledge that the number of manually annotated reports remains limited and may constrain broader external generalization. Nevertheless, the models demonstrated robust performance even under this small-sample regime, suggesting strong data efficiency and meaningful domain representation learning. Ongoing expert annotation efforts aim to expand the labeled corpus, enabling future cross-institutional validation and reducing performance variance in rare findings. Collectively, these results highlight a scalable and clinically interpretable approach for transforming unstructured radiology text into structured, actionable information for precision neurotrauma research and clinical decision support.

## 4 Methods

**Figure 1.**
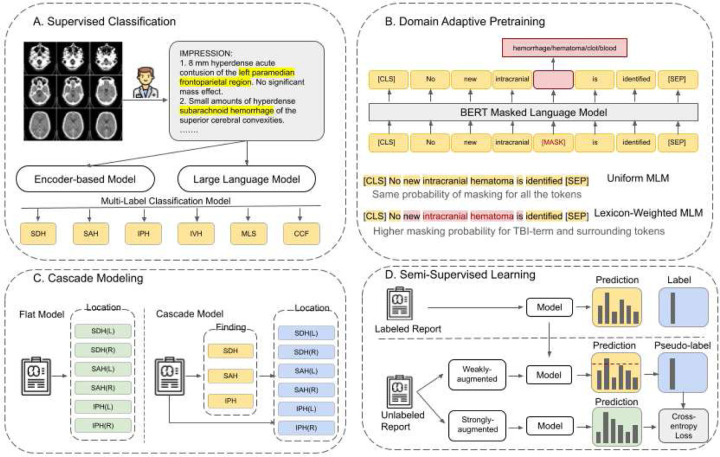
Integrated Framework for Radiology Report Analysis of Traumatic Brain Injury (TBI). A). *Supervised Classification*. Radiology reports are processed by both Encoder-based Models and Large Language Models. The model outputs are fed into a Multi-Label Classification Model to predict the presence of multiple TBI-related findings ( SDH: Subdural Hematoma, SAH: Subarachnoid Hemorrhage, IPH: Intraparenchymal Hemorrhage, IVH: Intraventricular Hemorrhage, MLS: Midline Shift, CFF: Calvarial/Facial Fracture). B). *Domain Adaptive Pretraining*: Specialized TBI knowledge is incorporated by continuing pre-training on a large corpus of head CT reports using the Masked Language Modeling (MLM) with Uniform probability and Lexicon-Weighted form that assigned a significantly higher masking probability to critical TBI terms and surrounding tokens. C). *Cascade Modeling*: Anatomical localization is performed using a two-stage cascade model and compare with the flat Model which predicts all finding-location pairs independently. D). *Semi-Supervised Learning*: A FixMatch-style strategy is used to leverage both labeled and unlabeled reports and trained to match the predictions between weakly-augmented and strongly-augmented views of the unlabeled data via a consistency regularization, gated by a prediction confidence threshold determined pseudo-labels.

**Figure 2.**
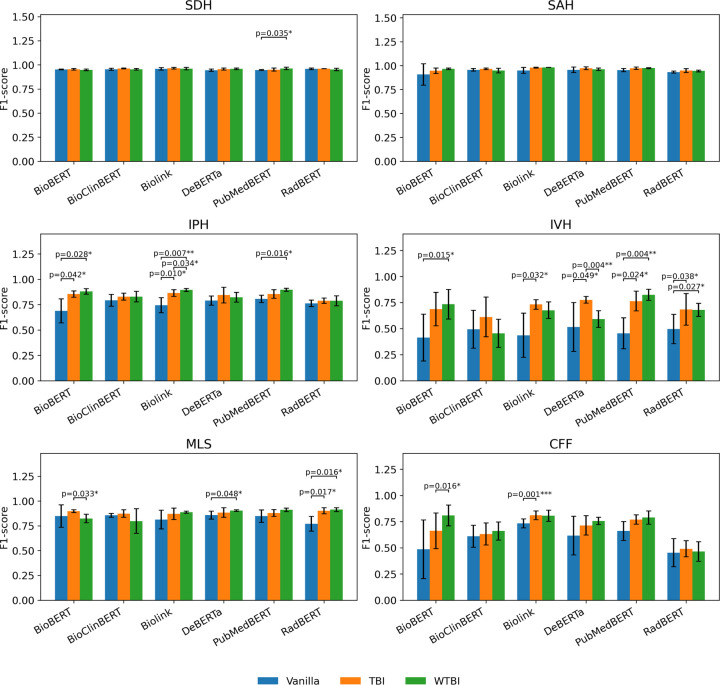
Comparison of Classification Performance for TBI Findings Using Different Encoder Architectures Following Domain-specific Pretraining on Radiology Reports. TBI: uniform domain-adaptive masked-language pretraining on TBI reports; WTBI: lexicon-weighted masked-language pretraining on TBI reports. SDH, subdural hemorrhage; SAH, subarachnoid hemorrhage; IPH, intraparenchymal hemorrhage; IVH, intraventricular hemorrhage; MLS, midline shift; CFF, cranial/facial fracture

**Figure 3.**
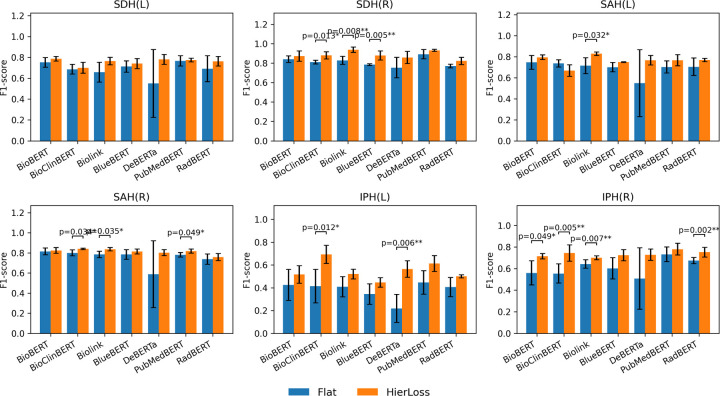
Comparison of Classification Performance for Location of TBI Findings Between Flat Models and Casecade Modeling with Hierachical Loss. SDH, subdural hemorrhage; SAH, subarachnoid hemorrhage; IPH, intraparenchymal hemorrhage; IVH, intraventricular hemorrhage; MLS, midline shift; CFF, cranial/facial fracture

**Figure 4.**
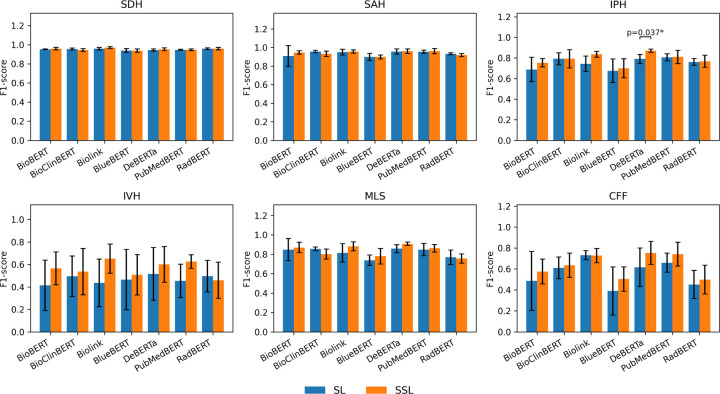
Comparison of Supervised and Semi-supervised Learning Approaches for TBI Findings Classification Using Various Encoder Architectures. SL: Supervised Learning; SSL: Semi-supervised Learning SDH, subdural hemorrhage; SAH, subarachnoid hemorrhage; IPH, intraparenchymal hemorrhage; IVH, intraventricular hemorrhage; MLS, midline shift; CFF, cranial/facial fracture

We designed an integrated framework for radiology report understanding that systematically evaluates model architectures, adaptation strategies, and learning paradigms in the TBI domain (Fig. 1). The overall workflow comprises four components: (A) supervised report classification using encoder-based models and generative LLMs; (B) domain-adaptive pretraining for corpus specialization; (C) cascade modeling for anatomical location inference; and (D) semi-supervised learning to leverage unlabeled data.

### 4.1 Supervised Classification Models

To classify TBI-related findings, we implemented multiple supervised text classifiers spanning encoder-based architectures and open-weight LLMs. Each model produces a contextual sequence embedding used by a multi-label classification head to predict individual findings.

#### Encoder-based models:

We evaluated seven encoder-based architectures including: BioBERT^20^, BioClinBERT^22^, BioLinkBERT^25^, BlueBERT^44^, DeBERTa^24^, PubMedBERT^21^, and RadBERT^23^. The final hidden state of the classification token provides the pooled representation:

(1) $$H_{\text{pooled}}=H_{\text{CLS}}.$$

#### Large language models:

We further evaluated Gemma^26^, Mistral^27^, LLaMA^28^, and Qwen^29^, and biomedical derivatives BioMistral^30^, Me-LLaMA^31^, MedAlpaca^32^. Parameter-Efficient Fine-Tuning (PEFT) via LoRA^33^ have been applied for efficient domain adaptation with limited computing resources. For LLMs, sequence-level embeddings were obtained via attention-masked mean pooling to exclude padding:

(2) $$H_{\text{pooled}}=\frac{\sum_{l=1}^{L}H_{l}⊙M_{l}}{\sum_{l=1}^{L}M_{l}},$$

where $H_{l}$ is the hidden state at position l, $M_{l}$ is the attention mask, and ⊙ denotes element-wise multiplication.

#### Supervised objective:

All models were trained for multi-label classification using binary cross-entropy with logits as the supervised loss function:

(3) $${𝓛}_{\sup}=-\frac{1}{2}\sum_{j=1}^{C}\left[y_{i}\log\sigma \left(z_{j}\right)+\left(1-y_{j}\right)\log\left(1-\sigma \left(z_{j}\right)\right)\right],$$

where $\sigma \left(\cdot \right)$ is the sigmoid function, $z_{j}$ is the logit for class j, $y_{j}\in \left\{0,1\right\}$ the ground-truth label, and C the number of classes.

### 4.2 Domain Pretraining Strategy

To specialize language representations to radiology narratives, we continued pretraining encoder-based models on the full head CT report corpus using masked language modeling (MLM). We assessed two configurations:

#### Uniform MLM:

Tokens were masked with equal probability, following the standard BERT setup. This method serves as a baseline for domain adaptation.

#### Lexicon-weighted MLM:

To emphasize clinically meaningful terms, we curated a TBI lexicon (see details in Table S5) with a four-fold higher masking probability for lexicon tokens and their 5 token neighborhood, encouraging richer representations around diagnostic terminology.

Each variant was initialized from public checkpoints and pretrained for 5 epochs, with maximum sequence length 512 and batch size of 8. Models adapted with uniform MLM are denoted *TBI-*, and those with lexicon-weighted MLM are *WTBI-*in the results section.

### 4.3 Cascade Model for Anatomical Localization

Anatomical location extraction was formulated as a two-stage cascade modeling that mirrors the clinical reasoning. Stage 1 detects each clinical finding (e.g., SDH, IVH); stage 2 predicts location (left/right/bilateral/none) conditional on a positive finding. This ensures location is inferred only when clinically meaningful. We optimized a weighted hierarchical objective:

(4) $${𝓛}_{\text{total}}={𝓛}_{\text{find}}+{\text{λ}}_{\text{loc}}\cdot {𝓛}_{\text{loc}},$$

where ${\text{λ}}_{\text{loc}}$ is a constant that balances identification and localization losses and enables end-to-end learning of context-aware spatial reasoning.

### 4.4 Semi-supervised Learning Setup

We adopted a FixMatch-style^40^ teacher–student approach with an exponential moving average (EMA) teacher and confidence-gated pseudo-labels over weak/strong textual augmentations. Labeled reports were optimized with ${𝓛}_{\sup}$ metioned above. For each unlabeled report u, we created a weak view $u^{\left(w\right)}$ (token dropout) and a strong view $u^{\left(s\right)}$ (lexicon-aware perturbations preserving clinical semantics, plus random span deletion, local shuffle, token dropout). The teacher produced soft targets on $u^{\left(w\right)}$, which served as pseudo-labels for the student on $u^{\left(s\right)}$ with temperature sharpening and distribution alignment (DA):

(5) $$\tilde{p}=\sigma \left(\frac{f_{\text{teacher}}\left(u^{\left(w\right)}\right)}{T}\right)$$

(6) $$p^{\text{DA}}=\text{clip}\left(\tilde{p}⊙\frac{\pi_{l}}{\pi_{u}+\varepsilon },0,\;1\right)$$

where T is the sharpening temperature, $\pi_{l}$ the running average of labeled class priors, $\pi_{u}$ the EMA of unlabeled predictions, and ⊙ element-wise multiplication. Hard pseudo-labels $\hat{y}$ and a confidence mask M were formed using per-class thresholds τ applied to $p^{\text{DA}}$. Given student logits $z^{\left(s\right)}=f_{\text{θ}}\left(u^{\left(s\right)}\right)$, the unsupervised loss is defined as:

(7) $${𝓛}_{\text{unsup}}\left(u\right)=\frac{\sum_{c=1}^{C}M_{c}\cdot \text{BCEWithLogits}\left(z_{c}^{\left(s\right)},{\hat{\text{y}}}_{c}\right)}{\sum_{c=1}^{C}M_{c}+\varepsilon }$$

### 4.5 Dataset

We curated de-identified head CT radiology reports from patients evaluated for TBI at the University of Michigan between January 2, 2015 and November 23, 2023. The study obtained approval from the Institutional Review Boards(IRB) of the University of Michigan Medical School (HUM00098656) and all research was performed in accordance with relevant guidelines/regulations. The dataset comprises 4,038 reports from 1,086 patients. Of these, 444 reports were annotated by an expert neurocritical care specialist for the presence of a critical finding that includes subdural hemorrhage (SDH), epidural hemorrhage (EDH), subarachnoid hemorrhage (SAH), intraparenchymal hemorrhage (IPH), intraventricular hemorrhage (IVH), cranial & facial fracture (CFF), and midline shift (MLS). For positive reports, laterality (left/right/bilateral) was recorded. Annotation details are summarized in Table S4. Although sourced from a single institution, the dataset encompasses patients referred to Michigan Medicine from across the state. The wide geographic coverage and involvement of numerous clinicians and radiologists contribute to substantial variability in reporting styles and clinical language, supporting the dataset’s representativeness despite its single-center origin.

#### Evaluation metrics and statistical analysis

We used 5-fold stratified cross-validation with a 20% hold-out test set unseen during training and validation. Performance was reported as per-class F1 and weighted F1 (mean ± standard deviation across folds). For class i,

(8) $$F1_{i}=\frac{2\cdot P_{i}\cdot R_{i}}{P_{i}+R_{i}},$$

(9) $$P_{i}=\frac{TP_{i}}{TP_{i}+FP_{i}},$$

(10) $$R_{i}=\frac{TP_{i}}{TP_{i}+FN_{i}}.$$

Weighted F1 aggregates classwise F1 using class supports $n_{i}$:

(11) $$F1_{\text{weighted}}=\frac{\sum_{\mathrm{i\in C}}n_{i}F1_{i}}{\sum_{\mathrm{i\in C}}n_{i}}.$$

Paired t-tests (two-sided) were conducted on fold-wise F1 for model comparisons. Bar plots show fold-aggregated metrics with standard deviation; significance is annotated as ∗ *P* < 0.05, ∗∗ *P* < 0.01, ∗ ∗ ∗ *P* < 0.001.

## Supplementary Material

Supplement 1

## Figures and Tables

**Table 1.** Performance of Encoder-based Findings Classification Models (F1-score) Results are reported as Mean (Standard Deviation) on the test set under a 5-fold cross-validation setting. SDH:Subdural Hemorrhage; SAH: Subarachnoid Hemorrhage; IPH:Intraparenchymal Hemorrhage; IVH:Intraventricular Hemorrhage; MLS:Midline Shift; CFF:Cranial&Facial Fracture TBI:Model pretrained using masked language modeling (MLM) on the TBI radiology report corpus. WTBI: Model pretrained using weighted MLM with increased sampling probability for TBI-related lexicon terms.

Model	SDH	SAH	IPH	IVH	MLS	CFF	Weighted
BioBERT	0.952 (0.004)	0.908 (0.111)	0.688 (0.119)	0.414 (0.224)	0.848 (0.113)	0.486 (0.28)	0.808 (0.086)
BioClinBERT	0.954 (0.011)	0.956 (0.013)	0.792 (0.058)	0.494 (0.181)	0.856 (0.019)	0.61 (0.104)	0.856 (0.026)
Biolink	0.958 (0.013)	0.95 (0.031)	0.744 (0.075)	0.436 (0.212)	0.814 (0.095)	0.732 (0.043)	0.848 (0.029)
BlueBERT	0.94 (0.02)	0.898 (0.039)	0.676 (0.115)	0.466 (0.269)	0.738 (0.054)	0.39 (0.231)	0.784 (0.055)
DeBERTa	0.944 (0.011)	0.956 (0.029)	0.79 (0.045)	0.516 (0.235)	0.858 (0.041)	0.616 (0.185)	0.852 (0.035)
PubMedBERT	0.946 (0.005)	0.954 (0.017)	0.806 (0.036)	0.454 (0.149)	0.848 (0.063)	0.66 (0.091)	0.854 (0.022)
RadBERT	0.958 (0.008)	0.932 (0.011)	0.762 (0.033)	0.496 (0.14)	0.77 (0.074)	0.452 (0.134)	0.824 (0.021)

TBI-BioBERT	0.954 (0.009)	0.946 (0.029)	0.854 (0.031)	0.688 (0.16)	0.898 (0.015)	0.662 (0.17)	0.884 (0.033)
TBI-BioClinBERT	0.962 (0.004)	0.966 (0.009)	0.828 (0.034)	0.612 (0.19)	0.874 (0.038)	0.632 (0.105)	0.876 (0.029)
TBI-Biolink	0.964 (0.009)	0.978 (0.004)	0.864 (0.033)	0.732 (0.046)	0.872 (0.058)	0.81 (0.042)	0.908 (0.011)
TBI-BlueBERT	0.96 (0)	0.96 (0.014)	0.848 (0.048)	0.65 (0.095)	0.88 (0.014)	0.49 (0.184)	0.87 (0.024)
TBI-DeBERTa	0.956 (0.011)	0.972 (0.015)	0.844 (0.077)	0.776 (0.033)	0.884 (0.048)	0.714 (0.092)	0.898 (0.016)
TBI-PubMedBERT	0.95 (0.016)	0.972 (0.013)	0.854 (0.042)	0.764 (0.095)	0.88 (0.035)	0.77 (0.045)	0.9 (0.017)
TBI-RadBERT	0.96 (0)	0.948 (0.019)	0.788 (0.026)	0.684 (0.152)	0.902 (0.03)	0.49 (0.077)	0.864 (0.018)

WTBI-BioBERT	0.948 (0.008)	0.966 (0.009)	0.88 (0.027)	0.734 (0.142)	0.824 (0.043)	0.808 (0.1)	0.898 (0.015)
WTBI-BioClinBERT	0.954 (0.009)	0.948 (0.025)	0.828 (0.052)	0.454 (0.135)	0.798 (0.124)	0.66 (0.086)	0.854 (0.03)
WTBI-Biolink	0.96 (0.012)	0.98 (0)	0.894 (0.015)	0.676 (0.08)	0.888 (0.011)	0.806 (0.054)	0.91 (0.012)
WTBI-BlueBERT	0.956 (0.011)	0.97 (0)	0.864 (0.043)	0.756 (0.038)	0.866 (0.022)	0.658 (0.06)	0.892 (0.008)
WTBI-DeBERTa	0.958 (0.008)	0.962 (0.013)	0.822 (0.048)	0.592 (0.08)	0.904 (0.009)	0.756 (0.036)	0.886 (0.011)
WTBI-PubMedBERT	0.962 (0.013)	0.974 (0.005)	0.896 (0.015)	0.824 (0.053)	0.912 (0.018)	0.788 (0.063)	0.92 (0.007)
WTBI-RadBERT	0.952 (0.013)	0.944 (0.009)	0.788 (0.049)	0.68 (0.063)	0.914 (0.019)	0.464 (0.093)	0.86 (0.014)

**Table 2.** Performance of Encoder-based Location Classification Models (F1-score) Results are reported as Mean (Standard Deviation) on the test set under a 5-fold cross-validation setting. SDH:Subdural Hemorrhage; SAH: Subarachnoid Hemorrhage; IPH:Intraparenchymal Hemorrhage; IVH:Intraventricular Hemorrhage Hier-:Model trained using hierarchical loss.

Model	SDH(L)	SDH(R)	SAH(L)	SAH(R)	IPH(L)	IPH(R)
BioBERT	0.752 (0.046)	0.84 (0.035)	0.746 (0.066)	0.814 (0.034)	0.424 (0.136)	0.56 (0.112)
BioClinBERT	0.686 (0.047)	0.81 (0.019)	0.736 (0.034)	0.8 (0.03)	0.414 (0.147)	0.554 (0.089)
Biolink	0.658 (0.095)	0.828 (0.041)	0.714 (0.075)	0.784 (0.032)	0.408 (0.089)	0.642 (0.039)
BlueBERT	0.712 (0.055)	0.784 (0.009)	0.7 (0.044)	0.784 (0.049)	0.344 (0.09)	0.602 (0.099)
DeBERTa	0.55 (0.325)	0.754 (0.106)	0.548 (0.317)	0.588 (0.333)	0.218 (0.123)	0.508 (0.285)
PubMedBERT	0.766 (0.049)	0.892 (0.048)	0.702 (0.058)	0.78 (0.022)	0.446 (0.104)	0.732 (0.068)
RadBERT	0.692 (0.125)	0.77 (0.019)	0.704 (0.083)	0.738 (0.052)	0.406 (0.084)	0.674 (0.029)

Hier-BioBERT	0.786 (0.022)	0.872 (0.054)	0.794 (0.023)	0.824 (0.028)	0.516 (0.077)	0.714 (0.025)
Hier-BioClinBERT	0.7 (0.052)	0.88 (0.037)	0.668 (0.055)	0.84 (0.007)	0.692 (0.079)	0.744 (0.076)
Hier-Biolink	0.764 (0.035)	0.938 (0.028)	0.828 (0.016)	0.836 (0.017)	0.52 (0.042)	0.7 (0.019)
Hier-BlueBERT	0.74 (0.048)	0.878 (0.046)	0.748 (0.004)	0.814 (0.025)	0.446 (0.042)	0.724 (0.051)
Hier-DeBERTa	0.78 (0.047)	0.858 (0.063)	0.766 (0.045)	0.802 (0.029)	0.564 (0.072)	0.728 (0.)
Hier-PubMedBERT	0.774 (0.018)	0.932 (0.011)	0.766 (0.053)	0.818 (0.02)	0.612 (0.07)	0.78 (0.055)
Hier-RadBERT	0.762 (0.045)	0.822 (0.037)	0.768 (0.016)	0.758 (0.034)	0.5 (0.014)	0.752 (0.045)

**Table 3.** Performance of Semi-supervised Encoder-based Findings Classification Models (F1-score) Results are reported as Mean (Standard Deviation) on the test set under a 5-fold cross-validation setting. SDH:Subdural Hemorrhage; SAH: Subarachnoid Hemorrhage; IPH:Intraparenchymal Hemorrhage; IVH:Intraventricular Hemorrhage; MLS:Midline Shift; CFF:Cranial&Facial Fracture

Model	SDH	SAH	IPH	IVH	MLS	CFF	Weighted
BioBERT	0.958 (0.013)	0.946 (0.018)	0.754 (0.04)	0.566 (0.146)	0.87 (0.055)	0.576 (0.118)	0.85 (0.019)
BioClinBERT	0.944 (0.015)	0.932 (0.029)	0.792 (0.088)	0.536 (0.207)	0.802 (0.051)	0.636 (0.115)	0.844 (0.027)
Biolink	0.97 (0.01)	0.956 (0.017)	0.836 (0.029)	0.652 (0.13)	0.882 (0.046)	0.728 (0.069)	0.888 (0.019)
BlueBERT	0.938 0.018)	0.898 (0.02)	0.7 (0.092)	0.508 (0.18)	0.78 (0.08)	0.504 (0.116)	0.804 (0.032)
DeBERTa	0.952 (0.015)	0.96 (0.025)	0.87 (0.016)	0.6 (0.159)	0.908 (0.018)	0.754 (0.11)	0.894 (0.015)
PubMedBERT	0.948 (0.008)	0.96 (0.028)	0.81 (0.066)	0.626 (0.059)	0.862 (0.04)	0.742 (0.113)	0.876 (0.019)
RadBERT	0.958 (0.013)	0.918 (0.016)	0.768 (0.058)	0.46 (0.161)	0.756 (0.048)	0.498 (0.137)	0.82 (0.039)

## Data Availability

The datasets used and/or analysed during the current study available from the corresponding author on reasonable request.
