# Supplementary material for: Leveraging Language Models for Automated Label Generation in Traumatic Brain Injury Radiology Reports
Source: Res Sq. 2025 Dec 4:rs.3.rs-8051129. Preprint. [Version 1] doi: 10.21203/rs.3.rs-8051129/v1 (PMC12687829; doi:10.21203/rs.3.rs-8051129/v1)
Supplement: Supplement 1 [file NIHPPrs8051129v1-supplement-1.pdf]

Model	Backbone	Pretraining Materials	Tasks	Parameters
BioBERT[20]	BERT-base(continual pre-training)	PubMed abstracts and PMC full-text articles	NER, RE and QA	110M
BioClinBERT[22]	BERT-base(continual pre-training from BioBERT)	Notes in the MIMIC-III database	NER	110M
BioLinkBERT[25]	BERT-base(continual pre-training from PubmedBERT)	PubMed articles with citation links	QA	110M
BlueBERT[44]	BERT-base	PubMed abstracts and clinical notes (MIMIC-III)	TC, NER, RE, SS	110M
DeBERTa[24]	BERT with disentangled attention	Books, Wikipedia, OpenWebText	NER, NLI, QA	135M
RadBERT[23]	RoBERTa-base	Radiology reports	Radiology-specific NLP tasks	110M
PubMedBERT[21]	Standard BERT architecture (from scratch)	PubMed abstracts	NER, RE, QA and DC	110M

Table S1. Summary of Finetuned Encoder-based Models for the Biomedical Domain.
TC:Text classification within medicine and biology; NER: Named Entity Recognition; NLI: Nature Language Inference; RE:Relation Extraction; QA: Question Answering; Radiology-specific NLP tasks include abnormal sentence classification, report coding, and report summarization

Supplementary Information

The following figures and tables are provided as supplementary material to support the main text.

Model	Backbone	Pretraining Materials	Tasks	Parameters
BioMistral[30]	Mistral-7B	PMC full-text articles	biomedical Question Answering and reasoning tasks based on scientific literature	7B
Gemma2[26]	Decoder-only Transformer	Web, Math, Code	Highly efficient NLU on edge deployment	2B
Llama-3.1[28]	Decoder-only Transformer	Publicly available online data.	Advanced reasoning, tool use, and multilingual capability	8B
MedAlpaca[32]	LLaMA Decoder	Medical QA and dialogue	Medical instruction-following and dialogue	8B
Me-LLaMA[31]	LLaMA2 Decoder	medical literature, clinical notes	clinical text analysis, summarization, and complex diagnostic reasoning	8B
Mistral[27]	Decoder-only Transformer	Web-scale, diverse open data	General reasoning, coding, text generation	7B
Qwen2.5[29]	Dense and MoE	Diverse data on knowledge, math, code, text generation and understanding	multilingual NLU and long-context summarization	2B

Table S2. Summary of Generative Large Language Models
NLU:Natural Language Understanding; MoE: Mixture of Experts Given that Large Language Models often exist across multiple parameter scales, we restrict the entries in this table to only the specific version evaluated in this research.

	25%	50%	75%	Mean
Number of CT scans/admission	2	3	4	3.78
Admission duration(Hour)	72	144	288	248
Order to exam time(Hour)	0.42	1.33	3.56	3.10
Exam to read time(Hour)	1.43	3.03	7.73	5.18
Impression word count	33	55	90	68
Narrative word count	153	205	301	247

Table S3. Summary Statistics of Radiology Report Metadata, Including CT Scan Frequency, Admission Duration, and Report Timing Metrics. Values represent the 25th, 50th (median), and 75th percentiles, along with the mean

	Findings	Location(Left)	Location(Right)
Subdural Hemorrhage(SDH)	339	206	230
Epidural Hemorrhage(EDH)	6	-	-
Subarachnoid Hemorrhage (SAH)	192	120	141
Intraparenchymal Hemorrhage (IPH)	163	86	108
Intraventricular Hemorrhage (IVH)	69	-	-
Midline Shift(MLS)	115	-	-
Cranial&Facial Fracture(CFF)	64	-	-
Total annotated reports	444	-	-

Table S4. Distribution of Annotated Findings and Their Lateralized Locations in Traumatic Brain Injury (TBI) Radiology Reports. The counts reflect the number of reports with each finding, including left- and right-sided annotations where applicable. A dash (-) indicates laterality information was not available or not applicable for that finding.

Finding	Synonyms
Cranial&Skull Fracture	skull fracture; calvarial fracture; displaced calvarial; bone fracture; fracture right; acute fracture; fracture involving; nondisplaced fracture; fractures involving; fracture visualized; displaced fracture; displaced fractures; comminuted fracture; maxillofacial fracture; orbital fracture
Epidural Hemorrhage	epidural hematoma; extra-axial hemorrhage; extra-axial hematoma; extra-axial blood; extra-axial clot
Hemorrhage	hemorrhage within; intracranial hemorrhage; acute intracranial; hemorrhage identified; hemorrhage noted; new hemorrhage
Intraparenchymal Hemorrhage	intraparenchymal hemorrhage; intraparenchymal hematoma; contusions; contusion; contusional; hemorrhagecontusion; hematomacontusion; contusionhematoma; swellingcontusion; hemorrhagecontusions; edemacontusion; contusionhemorrhage; intra axial
Intraventricular Hemorrhage	intraventricular hemorrhage; Hematoma within Ventricles; Hemorrhage within lateral ventricles; hemorrhage within occipital horns; hematoma within lateral ventricles; hematoma within occipital horns; layering hemorrhage in horns; layering hematoma in occipital horns; third ventricular hemorrhage; third ventricular hematoma
Subarachnoid Hemorrhage	subarachnoid hemorrhage; acute subarachnoid; subarachnoid blood; Subarachnoid Hematoma; Blood in Sulci; Blood in Fissure; Cisternal Hemorrhage; Basal Cisterns Hemorrhage; convexity hematoma; convexity hemorrhage; Sylvian fissure hemorrhage; Interhemispheric fissure hemorrhage; Sulcal hematoma; Sulcal Hemorrhage
Subdural hemorrhage	subdural hematoma; subdural hematomas; subdural fluid; convexity subdural; subfalcine herniation; acute subdural; extra axial; Convexity subdural; extra-axial; Parafalcine hemorrhage/hematoma/blood/clot; Tentorial hemorrhage; Blood along tentorium

Table S5. Custumized Tramatic Brain Injury Findings Lexicon

Model	SDH	SAH	IPH	IVH	MLS	CFF	Weighted
BioMistral	0.956 (0.011)	0.906 (0.018)	0.81 (0.027)	0.542 (0.24)	0.82 (0.06)	0.628 (0.143)	0.85 (0.032)
Llama-3.1-8B	0.948 (0.013)	0.914 (0.035)	0.848 (0.024)	0.662 (0.129)	0.81 (0.03)	0.684 (0.082)	0.866 (0.015)
Me-LLaMA	0.936 (0.032)	0.916 (0.023)	0.818 (0.046)	0.722 (0.053)	0.83 (0.066)	0.682 (0.097)	0.864 (0.015)
MedAlpaca	0.9 (0)	0.666 (0.043)	0.306 (0.038)	0 (0)	0.12 (0.059)	0 (0)	0.52 (0.012)
Meditron3	0.94 (0.007)	0.922 (0.015)	0.818 (0.026)	0.684 (0.193)	0.794 (0.125)	0.674 (0.101)	0.86 (0.021)
Mistral	0.944 (0.015)	0.93 (0.016)	0.826 (0.053)	0.626 (0.136)	0.866 (0.053)	0.676 (0.022)	0.868 (0.018)
Qwen2.5	0.956 (0.011)	0.928 (0.023)	0.806 (0.038)	0.69 (0.049)	0.872 (0.032)	0.562 (0.085)	0.862 (0.018)
Gemma2	0.936 (0.005)	0.926 (0.015)	0.794 (0.036)	0.654 (0.143)	0.786 (0.079)	0.68 (0.112)	0.85 (0.017)

Table S6. Performance of LLM Findings Classification Models (F1-score)
Results are reported as Mean (Standard Deviation) on the test set under a 5-fold cross-validation setting.
SDH:Subdural Hemorrhage; SAH: Subarachnoid Hemorrhage; IPH:Intraparenchymal Hemorrhage; IVH:Intraventricular Hemorrhage; MLS:Midline Shift; CFF:Cranial&Facial Fracture

Supplementary Files

This is a list of supplementary files associated with this preprint. Click to download.

- output.bbl
